# The Double Role of p53 in Cancer and Autoimmunity and Its Potential as Therapeutic Target

**DOI:** 10.3390/ijms17121975

**Published:** 2016-11-25

**Authors:** Alessandra Fierabracci, Marsha Pellegrino

**Affiliations:** Infectivology and Clinical Trials Area, Children’s Hospital Bambino Gesù, 00146 Rome, Italy; marsha.pellegrino@opbg.net

**Keywords:** p53, cancer, autoimmunity, p53 reactivation, novel therapeutic approaches

## Abstract

p53 is a sequence-specific short-lived transcription factor expressed at low concentrations in various tissues while it is upregulated in damaged, tumoral or inflamed tissue. In normally proliferating cells, p53 protein levels and function are tightly controlled by main regulators, i.e., MDM2 (mouse double minute 2) and MDM4 proteins. p53 plays an important role due to its ability to mediate tumor suppression. In addition to its importance as a tumor suppressor, p53 coordinates diverse cellular responses to stress and damage and plays an emerging role in various physiological processes, including fertility, cell metabolism, mitochondrial respiration, autophagy, cell adhesion, stem cell maintenance and development. Interestingly, it has been recently implicated in the suppression of autoimmune and inflammatory diseases in both mice and humans. In this review based on current knowledge on the functional properties of p53 and its regulatory pathways, we discuss the potential utility of p53 reactivation from a therapeutic perspective in oncology and chronic inflammatory disorders leading to autoimmunity.

## 1. Introduction

p53 (transformation-related protein 53) is part of a sequence-specific transcription factor family also including p63 and p73 [1]. The protein is expressed at low concentrations in various tissues while it is upregulated in damaged, tumoral or inflamed tissues [2,3,4,5]. Within the p53 molecule, a transactivation domain resides at the amino-terminus of the protein, a DNA-binding domain towards the middle section and an oligomerizationdomain at the carboxy-terminus. A portion of the COOH-terminus negatively regulates the central DNA binding site. p53 protein is a recognized short-lived transcription factor with important regulatory effects on the genome [6]. The effect of the protein is fulfilled by its action on several genes that regulate apoptosis and cell cycle arrest, as well as senescence, necroptosis, and others, in response to stress or genotoxic damage. A huge number of p53 target genes have been discovered in various cell types. Regarding the apoptotic pathway, Fas, death receptor 5, Bax (Bcl-2 associated X protein), PIG3 (p53-inducible gene 3), p85, PAG608 (p53 activated gene 608) and IGF-Bp3 (insulin-like growth factor binding protein 3) have been implicated [7,8,9,10,11,12,13,14,15,16,17]. p53 also intervenes in the regulation of NF-κB (nuclear factor kappa light chain enhancer of activated B cells) signaling [18] as applied to some transcription co-factors such as p300/CBP [19]. In addition, by inhibiting IKKs (IKB kinase) and histone H3 kinase, p53 suppresses NF-κB transcriptional activity [20]. Conversely, p53 is also able to induce the activation of NF-κB. Furthermore, loss of NF-κB function specifically abolishes the p53-mediated apoptotic response suggesting that has NF-κB an unexpected pro-apoptotic activity [21].

In normally proliferating cells, p53 is tightly controlled by the function of its main regulators, MDM2 (mouse double minute 2) and MDM4 proteins, which perform non-redundant activities [22]. Indeed, loss of MDM2 and MDM4 function in single knock-out mice (KO) leads to lethal activation of p53 and embryonic death at different stages of development [23,24,25]. MDM2 is an E3 ubiquitin ligase that ubiquitinates p53 and targets it to the proteasome for degradation. It also inhibits p53 transactivation activity [26,27]. MDM4 inhibits p53 transactivation activity as well but it is not able to ubiquitinate it [28]. However, through its binding to MDM2 it gives rise to a more effective ubiquitination executor [29,30,31]. Under basal growth conditions, this MDM2/MDM4 heterodimer is the functional inhibitor of p53 [29,30,31]. Following sublethal dose of DNA damage, MDM2, in addition to its role as p53 inhibitor, directs p53 activity towards the growth arrest response by downregulating proapoptotic cofactors [32]. Conversely, under severe conditions of DNA damage, MDM4 promotes p53-mediated mitochondrial apoptosis [33]. In agreement with these data, the lethality of the *mdm4* and *mdm2 KO* mice is accomplished by an apoptotic outcome in the *mdm2 KO* and mostly by a growth arrest response in the *mdm4 KO* [23,24,25]. The dissociation of MDM4 from MDM2 increases the activation of p53 whereas the association of MDM2 to MDM4 counteracts the proapoptotic activity of the last [34].

The role of p53 in human cancer has been the object of intensive investigations. Leading to evidence on the different mechanisms by which this protein mediates tumor suppression [35]. Recent data suggest emerging non-canonical functions of p53. In particular p53 regulates several cell programs including cell cycle arrest, non-canonical cell death mechanisms (apoptosis, autophagy) [36,37], metabolism [38], fertility [39], senescence [40], stem cell maintenance and development [41] and immune regulation [36]. p53 plays an emerging role in various physiological processes and coordinates diverse cellular responses to stress and damage [42]. In this manuscript we first review existing knowledge on the role of p53 in tumors to further discuss more recent insights on the ability of the protein to suppress inflammatory and autoimmune conditions both in humans and mice [36,43], thus opening up perspectives on the possible application of its reactivation mechanisms in the treatment of cancer, chronic inflammatory and autoimmune disorders.

## 2. p53 in Human Tumors

A variety of tumor suppressive mechanisms underlie the onset and progression of cancers. To inhibit tumor proliferation effectively, fine sensors are needed to single out the normal versus neoplastic growth [44]. p53 serves to selectively allow normal growth and inhibit the proliferation of the tumor thanks to its potent suppressive functions triggered only under damage or transforming conditions. Indeed, there is strong selection in almost all cancers against p53 activity [42].

As evidence of its protective role against tumor development, loss or mutation of p53 predisposes to a variety of spontaneous and induced tumors in animal models [35,45]. Most, if not all, human cancers negatively select for p53 function by either sporadic mutations in the p53 gene or alterations in genes that encode its main regulators [35,46,47,48]. Since p53 can prevent the malignant evolution of tumor cells, the tumor can regress and be cleared out by the reactivation of p53 activity in vivo. As stated above, p53 induction is crucial for tumor suppression throughout different biological responses, depending on the context, i.e., from growth arrest to senescence and autophagy [35,45,46,47,48]. It can also suppress tumor growth by switching on the major form of programmed cell death, apoptosis, which comprises specific and sequential events in the induction of cell death without inducing an inflammatory response [35]. In cultured cells, stress type and intensity, cell type and genetic background can be the triggering factors for the final response following p53 induction [35,49]. Moreover, crosstalks between other pathways can rebalance the decision versus either growth arrest or apoptosis. Furthermore, the anti-tumorigenic effect in different cell types can be exerted through several mechanisms [6].

**Figure 1:**
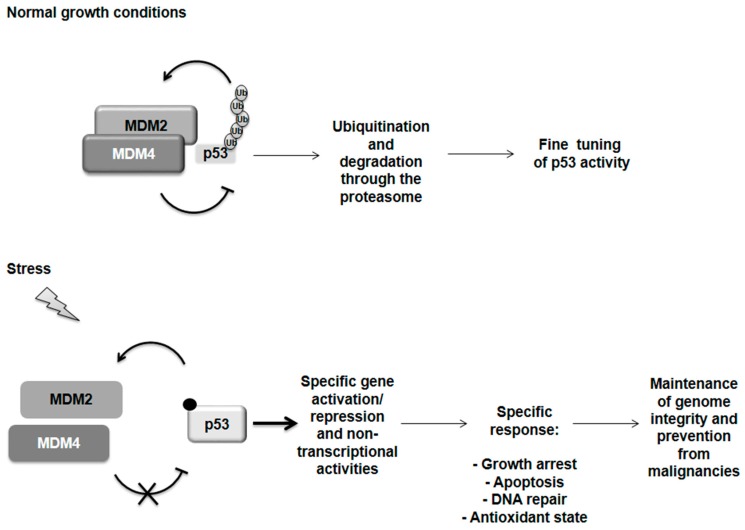
p53 regulation. Under normal growth conditions p53 is tightly controlled by its two main regulators, MDM2 and MDM4 proteins. Both can inhibit p53 transactivation activity. MDM2 is able to ubiquitinate p53 addressing it for degradation through the proteasome but it is sustained in this activity by the formation of a heterodimer with MDM4. Following severe stress, the MDM2 and MDM4 proteins dissociate by a still undefined mechanism while a variety of post-translational modifications are known for p53 (i.e., phosphorylation and acetylation). p53 is now free and active to orchestrate a response specifically related to the incoming stress and subsequently restore the normal condition by controlling its own regulator MDM2 in a negative feedback loop. However, if the damage is too severe, p53 can switch on programmed cell death in order to maintain genome integrity and prevent malignancies.

In those tumors that retain the wild-type p53 protein, its pathway is, however, abrogated, leading to aberrant p53 inactivation and avoidance of the tumor suppressor response [45]. These inactivating mechanisms include increased expression of the p53-negative regulators MDM2 and MDM4 [46,47,48] and deletion or epigenetic inactivation of the p53-positive regulator and MDM2 inhibitor ARF (Alternate Reading Frame tumor suppressor protein) [50,51]. On the other hand, in tumors that present a mutated p53 protein, usually located in the DNA binding domain, lead to impaired p53 transcriptional function and confer to the mutant protein a dominant negative activity over the remaining wild-type allele [52,53]. Moreover, many p53 mutants also gain new oncogenic properties [54]. These different situations require different therapeutic strategies to reactivate the p53 on cosuppressive response, currently under investigation [55] (Figure 1).

Regarding the different approaches to reactivate p53, whenever this protein is found as awild type in tumors and the p53-regulatory pathways are defective, the approach to its reactivation is the use of small molecules or peptides to inhibit the N-terminal interaction between p53 and MDM2 or MDM4. Most of these molecules induce p53 activation by disruption of its binding to MDM2, either acting in the p53 binding cleft of MDM2 (i.e., Nutlin, [56]) or by direct p53 targeting (i.e., RITA, [57]). Another study developed MDM4 inhibitors to be used in combination with those for MDM2 [58]. One of the drawbacks of these molecules is the inability to target both p53 regulators simultaneously. Therefore, recent studies attempted to develop dual MDM2/MDM4 inhibitors [44,59]. At last, the possibility to reactivate p53 in tumors by the use of a peptide that targets the MDM2/MDM4 heterodimer was exploited [60]. This approach allows the inhibition on p53 to be released more effectively.

Whenever p53 is mutated, such mutations lead to structural defects that prevent its appropriate folding, thus abolishing its binding to DNA. The ability of a molecule that acts as a chaperone to restore and stabilize the correct protein conformation was exploited (i.e., PRIMA-1, [61]). This approach was proven feasible based on the ability to correct misfoldings in other parts of p53 that are induced by the primary missense mutation [62]. Another attempt to restore the DNA-binding activity of p53 is the addition of zinc, used both in vitro and in xenograft models, an element important for the proper folding of the central core domain [63]. One additional approach was analyzed for those p53 mutations that result in early termination of translation to achieve bypassing of stop codons [44,64].

It must be emphasized that other strategies in the field of cancer therapeutics might be linked to the p53 pathway. One example is evident in the reactivation of the tumor suppressor retinoblastoma susceptibility gene (RB) through RB-inducing agents. RB protein plays an important role in faithful chromosome segregation, checkpoint control, apoptosis, senescence and terminal differentiation. This protein suppresses tumor formation by means of its multiple biological functions. Moreover, RB has been shown to bind to MDM2, the negative regulator of p53. By doing so, RB positively regulates p53 apoptotic activity [65]. Another example is the treatment of non-small cell lung cancer with the homeopathic remedy sulphur. This strategy leads to the activation of p53, which in turns competes with NF-κB for the binding to p300. The favored p53–p300 complex antagonizes the anti-apoptotic activity of NF-κB-p300 complex by the activation of apoptosis-inducing Bax factor [66].

## 3. p53 in Inflammatory and Autoimmune Conditions

The role of p53 in inflammation has been extensively in experimental animal models especially in reference to non-organ specific autoimmune diseases [36,43,67,68,69]. Systemic p53-deficient mice develop more rapidly collagen-induced arthritis and antigen-induced arthritis [70,71]. These strains are also more susceptible to developing experimental autoimmune encephalomyelitis and streptozotocin (STZ)-induced diabetes in respect to control wild type mice [72,73]. Systemic p53-deficient CD45.1 mice also develop a disorder similar to glomerulonephritis with high titers of proinflammatory cytokines [74].

As regards to organ-specific autoimmune diseases, the putative role of the p53 protein has been investigated in insulin-dependent diabetes (Type 1 diabetes, T1D) [73]. In more detail Zheng et al. (2005) [73] evaluated the role of the protein in regulating the autoimmune process in low-dose STZ-p53 deficient C57BL/6 mice. They showed that this strain produces higher levels of proinflammatory cytokines interleukin (IL-) 1, 6 and 12. Furthermore *p53^−/−^* macrophages exhibited a higher innate immune response to lipopolysaccharide (LPS) and interferon γ (IFN γ) compared to *p53^+/+^* mice. They provide evidence that p53 deficiency increases the incidence of T1D caused by p53-mediated inhibition of proinflammatory cytokines and of total and phosphorylated signal transducer and activator of transcription STAT-1.

In the light of the foregoing, the mechanism by which p53 is involved in the suppression of autoimmunity development remains to be further elucidated. While unravelling this issue, we have to bear in mind that, as for many transcription factors, the net effect of the protein comes from the balance of its expression in cells of the immune system as well as in non-immune cells such as the pancreatic islets in T1D. As regards several functionally distinct peripheral T cell subpopulations, they play a pivotal role in the control of the immunological response, i.e., effector, regulatory and memory T cells. In normal conditions, T regulatory cells (Treg) are present in the peripheral blood where they represent approximately 2%–5% of the total pool. FoxP3 (forkhead box P3) Treg originate in the thymus as a naturally arising subset and in the peripheral compartment as an inducible population [75]. Treg play an important role in maintaining immunological tolerance and homeostasis within the lymphocyte compartment and preventing autoimmunity onset by antagonizing the effect of autoreactive T cells. As opposite to the phenomenon of thymic negative selection of self-antigen reactive or autoreactive T cells, Treg exert in a immunosuppression dominant manner [75,76] throughout secretion of contraregulatory cytokines (i.e., IL-10 and IL-35), affecting or eliminating antigen-presenting cells (APC) and deprivation of cytokines [77,78,79,80]. Interestingly Kawashima et al. [67] attempted to investigate the mechanism of p53 suppression of autoimmunity development in p53-deficient mice *CD4-Cre p53 fl*/*fl* mice or p53 conditional KO mice. They found that aged *p53*/*cKO* mice spontaneously developed several inflammatory diseases such as thyroiditis, sialoadenitis, interstitial pneumonitis, hepatis, gastritis and glomerulonephritis. Moreover, they observed that in *p53/KO* mice the development of inflammatory lesions was paralleled by the reduction of FoxP3 CD4+CD25+ Treg. These data strongly support the putative effect of p53 in inducing the expression of FoxP3, i.e., a master regulator of Treg, by binding to the promoter and the conserved noncoding DNA sequence-2 of the *Foxp3* gene. Interestingly, p53’s effects appeared to be on the development and maintenance of Treg rather than on their suppressive function [67].

Of note, Park et al. [68] investigated the connection between p53 and IL-17/Treg cell balance in DBA/1J and C57BL/6 mice. They observed that CD4+ T cells from *p53^−/−^* mice decreased the activity of signal transducer and activator of transcription 5 (STAT-5), lowered the level of STAT-5 and compromised Treg cell differentiation. CD4+ T cells from *p53^−/−^* mice were less prone to differentiate toward Treg expressing lower FoxP3 levels. They also demonstrated that p53 regulates Treg cell differentiation by interacting directly with STAT-5. In inflammatory conditions p53 suppressed Th17 cell differentiation skewing T cells toward Treg through STAT-5 signaling. In mice affected by collagen-induced arthritis, administration of a p53 overexpression vector or an antagonist of MDM2 controlled arthritis development [68]. Consistently in rheumatoid arthritis (RA) patients, inhibition of p53-MDM2 interaction up-regulated Treg [68].

Interestingly, activation-induced cell death (AIDC) of T lymphocytes is a recognized mechanism of immune regulation ensuring the control of the size of the activated pool of T cells subsequent to its expansion [81]. Caspase activation, Fas (CD95) expression and IL-2 are critical in AIDC [82,83]. This phenomenon appeared to be independent of p53, although consistently, p53-deficient mice activated T cells were equally sensitive to AIDC as control mice [84]. Singh et al. (2010) [85] observed a non-classical process of antigen-induced T cell death in CD4+CD25− and CD8+ T cells by sustained T cell receptor (TCR) stimulation caused by plate-bound anti-CD3/anti-CD28 antibodies (Abs) which is dependent on p53. The last contributes to cell death of CD4+CD25− cells via upregulation of Fas. Remarkably, naturally occurring FoxP3+ Treg were found resistant to this form of apoptosis undergoing significant expansion upon stimulation [85]. Watanabe et al. (2014) [86,87] highlighted, still in a mouse model, that downmodulation of p53 following TCR signaling enforces antigen-specific CD4+ T cell responses while limiting bystander proliferation of non-specific T cells. Instead, in absence of TCR signaling, IL-2 induces sustained T cell proliferation.

Several studies also document the effect of p53 in human experimental models of inflammation. NK-κB and p53, known to exert opposite effects in cancer cells, coregulate proinflammatory cytokine secretion in primary human monocytes and macrophages, thus modulating their control on tissue microenvironment [88]. A novel p53-dependent enhancement of IFN (interferon) signaling through the induction of genes containing IFN-stimulated response elements was demonstrated in virally infected human cell lines, indicating that p53 could lead to an inhibitory effect on early virus replication [89]. A role of p53 has been documented in the regulation of Toll-like receptor 3 (TLR3) expression and function in human epithelial cell lines [90]. This suggests the existence of a positive feedback loop between p53, TLR3 and IFN-beta (β) in anti-viral defenses and a putative p53 role in the modulation of innate immune and antiviral responses. Genotoxic drugs were found to activate a novel STAT1 pathway dependent on p53 in human cell lines, thus sensitizing cells to IFN response [91]. p53 and its target p53-upregulated modulator of apoptosis (PUMA) were found to upregulate independently apoptosis of intestinal epithelial cells in patients and mice affected by colitis [92]. In human settings aAbs to the C-terminal domain of p53 were detected in the sera of patients affected by systemic lupus erythematosus (SLE) [93] responsible for inhibition of the protein function. p53 autoAbs were also reported in the sera of patients with systemic sclerosis (SS) [94] and Sjὃgren syndrome [95]. As opposite to the above mentioned studies in the Caucasian population, absence of anti-p53 Abs was noted in Chinese patients with SLE and RA [96]. Overexpression of TATA binding protein (TBP) and p53 as well as corresponding autoAbs were detected in the serum of Asian Indian patients affected by SLE, overlap syndromes including mixed connective tissue diseases (MCTD) and SS [97] possibly due to the hyperactivation of regulatory regions in these genes. High prevalence of anti-p53 Abs was also depicted in Japanese patients with dermatomyositis/polymyositis [98]. In patients positive for anti-p53 Abs immunoglobulin G levels were significantly higher [98]. Anti-p53 Abs were found to mimic damaged DNA immunologically [99]. High titers of anti-p53 Abs were also depicted in patients affected by Graves’ disease and immune vasculitis including Wegener’s granulomatosis [100]. 23% was the overall prevalence corresponding to that of cancer patients.

Gene expression microarrays analysis of patients with benign multiple sclerosis (BMS) revealed a signature with 30 p53 target genes. Among these, 19 had expression consistent with activation of p53 protein [101]. Furthermore, p53 gene mutations have been discovered in synovial cells of patients affected by RA [102,103]. Mutants of p53 and subsequent selection of mutant synoviocytes would not be causative of RA but could contribute to disease progression and perpetuation. Mutants N239S and R213 can be dominant negative and suppress endogenous wild-type protein, thus altering the function of synovial cells [104]. A TP53 codon 72 Arg/Arg polymorphism was found associated with a higher risk for inflammatory bowel disease development [105].

## 4. Cancer and Autoimmunity: p53 Probing the Link

In recent years, longitudinal observational studies have highlighted a bidirectional and dynamic link between cancer and autoimmunity, especially for non-organ-specific rheumatic diseases [106]. The association of thyroid autoimmunity and breast cancer has also been reported [107]. Chronic inflammation, damage, or effects of treatments may lead to malignant transformation or immune responses generated against tumor growth and can further result in autoimmune progression. In the light of this, there is an increased need to define best strategies to screen for, diagnose and treat these associated conditions.

The underlying reason for this increased risk remains to be elucidated at the molecular level although in some patients a common inciting trigger may act for both cancer and “paraneoplastic” autoimmune disease [108].

Recent evidences further support the double role of p53 in cancer and autoimmunity. As regards the expression of FoxP3, as master regulator suppressing breast cancer oncogenes *Skp2* (S-phase kinase associated protein 2) and *ErbB2* (epidermal growth factor receptor 2) involved in cancer progression, was detected in p53-dependent DNA damage responses in human breast and colon cancer cells [109]. In the context of tumor suppression FoxP3 induction was found regulated via a p53-dependent manner upon DNA damage.

Remarkably, to further unravel this issue, it is interesting to observe the secondary autoimmune responses detected in cancer patients. In relation to this phenomenon, p53 Abs were detected in the sera of patients affected by pancreatic adenocarcinoma, serous ovarian and breast cancer [110]. These have been proposed as potential biomarkers for early detection and prognosis [110,111]. In the other studies, immunoreactivity against MDM2 and p53 has also been envisaged as biomarker for the immunodiagnosis of esophageal squamous cell carcinoma [112].

## 5. Conclusive Remarks: Therapeutic Perspectives Based on p53 Reactivation

Discovery of new molecular targets for treatment of pathological conditions at increased incidence worldwide is an area in which considerable efforts are being employed to develop new and safe therapeutic agents [60]. In the light of the aforementioned acquired knowledge and discussion on the functional properties of p53 and its regulatory pathways, this protein can offer a focused target for future therapeutic perspectives in oncology and chronic inflammatory disorders that lead to autoimmunity. We also need to highlight that the co-occurrence of autoimmune responses in cancer patients or the presentation of associated autoimmune diseases and cancer [107] may putatively suggest the double-utility of p53 reactivation as therapeutic goal. More in detail, pharmacological blockade of the interaction between p53 and its crucial inhibitor MDM2 has been an attractive pipeline for tumors expressing wild-type p53 [44]. The NF-κB antagonist and p53-agonist activities of MDM2 inhibitors may have potential therapeutic effects by blocking the autoreactive T and B cell responses in systemic autoimmune disorders such as SLE.

**Figure 2:**
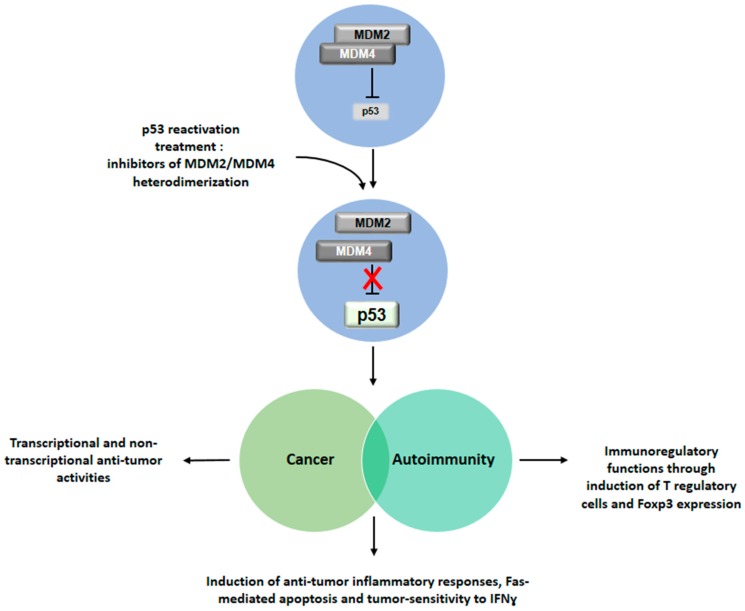
Implications of p53 reactivation for the treatment of cancer, autoimmunity and their associated conditions. Drug-induced inhibition of the MDM2/MDM4 heterodimer leads to reactivation of p53 function. Restored p53 activity would exhibit an anti-tumor role in cancer, an immunomodulatory activity in the context of autoimmune diseases and increased inflammatory immune response against tumor. This double effect could be exerted in patients affected by associated conditions of cancer and autoimmunity.

Recently, in addition to reactivating p53 by targeting the interface between the protein and MDM2 or MDM4, novel strategies have been proposed that interfere with MDM2/MDM4 heterodimerization [60] (Figure 2). These can also open up new pathways for treatment of autoimmunity and associated conditions of cancer and autoimmune diseases. This new pipeline for treatment may not only act on the transcriptional and non-transcriptional anti-tumor activities, but can also be beneficial for the induction of anti-tumor inflammatory responses, Fas-mediated apoptosis and tumor-sensitivity to IFNγ. With special reference to autoimmunity, the immunomodulatory treatment may function through induction of Treg and Foxp3 expression at diagnosis and during long-term disease to control disease development in association with hormonal replacement therapy in case of endocrine autoimmune disorders but also with specific treatments applied to non-organ-specific conditions.

Nevertheless, as for every new area of drug discovery, safety and efficacy concerns must be addressed both in preclinical and clinical settings, especially when a certain cell type has to be targeted and/or a biological effect is desired. Side effects can be produced by inappropriate dosing, such as suppressive effects on wound healing or tissue repair following toxic or ischemic damage or impaired host defense responses [69]. Thus, appropriate dosing of the drug is necessary to control intensity and duration of effect thus to warrant a desired level of p53 activity.

In the light of the foregoing, activation or restoring of p53 pathway has already reached the step of clinical trial exploitation [44]. As regards small molecule inhibitors of p53-MDM2 interaction or p53, drugs that act as chaperones in correcting a dysfunctional p53 pathway are under investigation [44]. For example, Nutlin [56] has been developed to target the p53-MDM2 interaction. Ongoing Phase I trials are selectively evaluating safety and efficacy of p53-MDM2 interaction including RG7112 (Roche) or MI-773 (Sanofi) or DS-3032b (Daiicgi-Sankyo). These represent initial milestones of a promising research pipeline for therapy.

## References

[1] Levrero M., de Laurenzi V., Costanzo A., Gong J., Wang J.Y., Melino G.J. (2000). The p53/p63/p73 family of transcription factors: Overlapping and distinct functions. Cell Sci..

[2] Arrowsmith C.H. (1999). Structure and function in the p53 family. Cell Death Differ..

[3] Yamanishi Y., Boyle D.L., Pinkoski M.J., Mahboubi A., Lin T., Han Z., Zvaifler N.J., Green D.R., Firestein G.S. (2002). Regulation of joint destruction and inflammation by p53 in collagen-induced arthritis. Am. J. Pathol..

[4] Sakhi S., Bruce A., Sun N., Tocco G., Baudry M., Schreiber S.S. (1994). p53 induction is associated with neuronal damage in the central nervous system. Proc. Natl. Acad. Sci. USA.

[5] Moon C., Kim S., Wie M., Kim H., Cheong J., Park J., Jee Y., Tanuma N., Matsumoto Y., Shin T. (2000). Increased expression of p53 and Bax in the spinal cords of rats with experimental autoimmune encephalomyelitis. Neurosci. Lett..

[6] Green D.R., Kroemer G. (2009). Cytoplasmic functions of the tumour suppressor p53. Nature.

[7] Wu G.S., Burns T.F., McDonald E.R., Jiang W., Meng R., Krantz I.D., Kao G., Gan D.D., Zhou J.Y., Muschel R. (1997). KILLER/DR5 is a DNA damage-inducible p53-regulated death receptor gene. Nat. Genet..

[8] Guan B., Yue P., Clayman G.L., Sun S.Y. (2001). Evidence that the death receptor DR4 is a DNA damage-inducible, p53-regulated gene. J. Cell Physiol..

[9] Müller M., Wilder S., Bannasch D., Israeli D., Lehlbach K., Li-Weber M., Friedman S.L., Galle P.R., Stremmel W., Oren M. (1998). p53 activates the CD95 (APO-1/Fas) gene in response to DNA damage by anticancer drugs. J. Exp. Med..

[10] Bennett M., Macdonald K., Chan S.W., Luzio J.P., Simari R., Weissberg P. (1998). Cell surface trafficking of Fas: A rapid mechanism of p53-mediated apoptosis. Science.

[11] Polyak K., Xia Y., Zweier J.L., Kinzler K.W., Vogelstein B. (1997). A model for p53-induced apoptosis. Nature.

[12] Buckbinder L., Talbott R., Velasco-Miguel S., Takenaka I., Faha B., Seizinger B.R., Kley N. (1995). Induction of the growth inhibitor IGF-binding protein 3 by p53. Nature.

[13] El-Deiry W.S., Harper J.W., O’Connor P.M., Velculescu V.E., Canman C.E., Jackman J., Pietenpol J.A., Burrell M., Hill D.E., Wang Y. (1994). WAF1/CIP1 is induced in p53-mediated G1 arrest and apoptosis. Cancer Res..

[14] Hermeking H., Lengauer C., Polyak K., He T.C., Zhang L., Thiagalingam S., Kinzler K.W., Vogelstein B. (1997). 14-3-3sigma is a p53-regulated inhibitor of G2/M progression. Mol. Cell.

[15] Zhan Q., Fan S., Bae I., Guillouf C., Liebermann D.A., O’Connor P.M., Fornace A.J. (1994). Induction of bax by genotoxic stress in human cells correlates with normal p53 status and apoptosis. Oncogene.

[16] Utrera R., Collavin L., Lazarević D., Delia D., Schneider C. (1998). A novel p53-inducible gene coding for a microtubule-localized protein with G2-phase-specific expression. EMBO J..

[17] Gudkov A.V., Gurova K.V., Komarova E.A. (2011). Inflammation and p53: A tale of two stresses. Genes Cancer.

[18] Ikeda A., Sun X., Li Y., Zhang Y., Eckner R., Doi T.S., Takahashi T., Obata Y., Yoshioka K., Yamamoto K. (2000). p300/CBP-dependent and -independent transcriptional interference between NF-κB RelA and p53. Biochem. Biophys. Res. Commun..

[19] Gu L., Zhu N., Findley H.W., Woods W.G., Zhou M. (2004). Identification and characterization of the IKKα promoter: Positive and negative regulation by ETS-1 and p53, respectively. J. Biol. Chem..

[20] Kawauchi K., Araki K., Tobiume K., Tanaka N. (2008). Activated p53 induces NF-κB DNA binding but suppresses its transcriptional activation. Biochem. Biophys. Res. Commun..

[21] Ryan K.M., Ernst M.K., Rice N.R., Vousden K.H. (2000). Role of NF-κB in p53-mediated programmed cell death. Nature.

[22] Marine J.C., Francoz S., Maetens M., Wahl G., Toledo F., Lozano G. (2006). Keeping p53 in check: Essential and synergistic functions of MDM2 and MDM4. Cell Death Differ..

[23] Parant J., Chavez-Reyes A., Little N.A., Yan W., Reinke V., Jochemsen A.G., Lozano G. (2001). Rescue of embryonic lethality in MDM4-null mice by loss of Trp53 suggests a non-overlapping pathway with MDM2 to regulate p53. Nat. Genet..

[24] Migliorini D., Danovi D., Colombo E., Carbone R., Pelicci P.G., Marine J.C. (2002). Hdmx recruitment into the nucleus by HDM2 is essential for its ability to regulate p53 stability and transactivation. J. Biol. Chem..

[25] Finch R.A., Donoviel D.B., Potter D., Shi M., Fan A., Freed D.D., Wang C.Y., Zambrowicz B.P., Ramirez-Solis R., Sands A.T. (2002). MDMX is a negative regulator of p53 activity in vivo. Cancer Res..

[26] Barak Y., Juven T., Haffner R., Oren M. (1993). MDM2 expression is induced by wild type p53 activity. EMBO J..

[27] Wu X., Bayle J.H., Olson D., Levine A.J. (1993). The p53-MDM-2 auto-regulatory feedback loop. Genes Dev..

[28] Shvarts A., Steegenga W.T., Riteco N., van Laar T., Dekker P., Bazuine M., van Ham R.C., van der Houven van Oordt W., Hateboer G., van der Eb A.J. (1996). MDMX: A novel p53-binding protein with some functional properties of MDM2. EMBO J..

[29] Linares L.K., Hengstermann A., Ciechanover A., Müller S., Scheffner M. (2003). HDMX stimulates HDM2-mediated ubiquitination and degradation of p53. Proc. Natl. Acad. Sci. USA.

[30] Linke K., Mace P.D., Smith C.A., Vaux D.L., Silke J., Day C.L. (2008). Structure of the MDM2/MDMX RING domain heterodimer reveals dimerization is required for their ubiquitylation in trans. Cell Death Differ..

[31] Okamoto K., Taya Y., Nakagama H. (2003). MDMX enhances p53 ubiquitination by altering the substrate preference of the MDM2 ubiquitin ligase. FEBS Lett..

[32] Shmueli A., Oren M. (2007). MDM2: p53’s lifesaver?. Mol. Cell.

[33] Mancini F., Di Conza G., Pellegrino M., Rinaldo C., Prodosmo A., Giglio S., D’Agnano I., Florenzano F., Felicioni L., Buttitta F. (2009). MDM4 (MDMX) localizes at the mitochondria and facilitates the p53-mediated intrinsic-apoptotic pathway. EMBO J..

[34] Mancini F., Pieroni L., Monteleone V., Lucà R., Fici L., Luca E., Urbani A., Xiong S., Soddu S., Masetti R. (2016). MDM4/HIPK2/p53 cytoplasmic assembly uncovers coordinated repression of molecules with anti-apoptotic activity during early DNA damage response. Oncogene.

[35] Meek D.W. (2009). Tumour suppression by p53: A role for the DNA damage response. Nat. Rev. Cancer.

[36] Muñoz-Fontela C., Mandinova A., Aaronson S.A., Lee S.W. (2016). Emerging roles of p53 and other tumour-suppressor genes in immune regulation. Nat. Rev. Immunol..

[37] Maiuri M.C., Galluzzi L., Morselli E., Kepp O., Malik S.A., Kroemer G. (2010). Autophagy regulation by p53. Curr. Opin. Cell Biol..

[38] Berkers C.R., Maddocks O.D., Cheung E.C., Mor I., Vousden K.H. (2013). Metabolic regulation by p53 family members. Cell Metab..

[39] Levine A.J., Tomasini R., McKeon F.D., Mak T.W., Melino G. (2011). The p53 family: Guardians of maternal reproduction. Nat. Rev. Mol. Cell Biol..

[40] Qian Y., Chen X. (2013). Senescence regulation by the p53 protein family. Methods Mol. Biol..

[41] Insinga A., Cicalese A., Pelicci P.G. (2014). DNA damage response in adult stem cells. Blood Cells Mol. Dis..

[42] Junttila M.R., Evan G.I. (2009). p53—A Jack of all trades but master of none. Nat. Rev. Cancer.

[43] Takatori H., Kawashima H., Suzuki K., Nakajima H. (2014). Role of p53 in systemic autoimmune diseases. Crit. Rev. Immunol..

[44] Khoo K.H., Verma C.S., Lane D.P. (2014). Drugging the p53 pathway: Understanding the route to clinical efficacy. Nat. Rev. Drug Discov..

[45] Brown C.J., Cheok C.F., Verma C.S., Lane D.P. (2010). Reactivation of p53: From peptides to small molecules. Trends Pharmacol. Sci..

[46] Danovi D., Meulmeester E., Pasini D., Migliorini D., Capra M., Frenk R., de Graaf P., Francoz S., Gasparini P., Gobbi A. (2004). Amplification of MDMX (or MDM4) directly contributes to tumor formation by inhibiting p53 tumor suppressor activity. Mol. Cell. Biol..

[47] Laurie N.A., Donovan S.L., Shih C.S., Zhang J., Mills N., Fuller C., Teunisse A., Lam S., Ramos Y., Mohan A. (2006). Inactivation of the p53 pathway in retinoblastoma. Nature.

[48] Riemenschneider M.J., Büschges R., Wolter M., Reifenberger J., Boström J., Kraus J.A., Schlegel U., Reifenberger G. (1999). Amplification and overexpression of the MDM4 (MDMX) gene from 1q32 in a subset of malignant gliomas without TP53 mutation or MDM2 amplification. Cancer Res..

[49] Murray-Zmijewski F., Slee E.A., Lu X. (2008). A complex barcode underlies the heterogeneous response of p53 to stress. Nat. Rev. Mol. Cell Biol..

[50] Esteller M., Cordon-Cardo C., Corn P.G., Meltzer S.J., Pohar K.S., Watkins D.N., Capella G., Peinado M.A., Matias-Guiu X., Prat J. (2001). p14ARF silencing by promoter hypermethylation mediates abnormal intracellular localization of MDM2. Cancer Res..

[51] Sherr C.J., Weber J.D. (2000). The ARF/p53 pathway. Curr. Opin. Genet. Dev..

[52] Milner J., Medcalf E.A. (1991). Cotranslation of activated mutant p53 with wild-type p53 protein into the mutant conformation. Cell.

[53] Sigal A., Rotter V. (2000). Oncogenic mutations of the p53 tumor suppressor: The demons of the guardian of the genome. Cancer Res..

[54] Levine A.J., Wu M.C., Chang A., Silver A., Attiyeh E.F., Lin J., Epstein C.B. (1995). The spectrum of mutations at the p53 locus. Evidence for tissue-specific mutagenesis, selection of mutant alleles, and a “gain of function” phenotype. Ann. N. Y. Acad. Sci..

[55] Ribeiro C.J., Rodrigues C.M., Moreira R., Santos M.M. (2016). Chemical variations on the p53 reactivation theme. Pharmaceuticals.

[56] Vassilev L.T., Vu B.T., Graves B., Carvajal D., Podlaski F., Filipovic Z., Kong N., Kammlott U., Lukacs C., Klein C. (2004). In vivo activation of the p53 pathway by small-molecule antagonists of MDM2. Science.

[57] Issaeva N., Bozko P., Enge M., Protopopova M., Verhoef L.G., Masucci M., Pramanik A., Selivanova G. (2004). Small molecule RITA binds to p53, blocks p53-HDM-2 interaction and activates p53 function in tumors. Nat. Med..

[58] Reed D., Shen Y., Shelat A.A., Arnold L.A., Ferreira A.M., Zhu F., Mills N., Smithson D.C., Regni C.A., Bashford D. (2010). Identification and characterization of the first small molecule inhibitor of MDMX. J. Biol. Chem..

[59] Wade M., Wahl G.M. (2009). Targeting MDM2 and MDMX in cancer therapy: Better living through medicinal chemistry?. Mol. Cancer Res..

[60] Pellegrino M., Mancini F., Lucà R., Coletti A., Giacchè N., Manni I., Arisi I., Florenzano F., Teveroni E., Buttarelli M. (2015). Targeting the MDM2/MDM4 interaction interface as a promising approach for p53 reactivation therapy. Cancer Res..

[61] Bykov V.J., Issaeva N., Shilov A., Hultcrantz M., Pugacheva E., Chumakov P., Bergman J., Wiman K.G., Selivanova G. (2002). Restoration of the tumor suppressor function to mutant p53 by a low-molecular-weight compound. Nat. Med..

[62] Nikolova P.V., Wong K.B., DeDecker B., Henckel J., Fersht A.R. (2000). Mechanism of rescue of common p53 cancer mutations by second-site suppressor mutations. EMBO J..

[63] Puca R., Nardinocchi L., Porru M., Simon A.J., Rechavi G., Leonetti C., Givol D., D’Orazi G. (2011). Restoring p53 active conformation by zinc increases the response of mutant p53 tumor cells to anticancer drugs. Cell Cycle.

[64] Petitjean A., Mathe E., Kato S., Ishioka C., Tavtigian S.V., Hainaut P., Olivier M. (2007). Impact of mutant p53 functional properties on TP53 mutation patterns and tumor phenotype: Lessons from recent developments in the IARC TP53 database. Hum. Mutat..

[65] Hsieh J.K., Chan F.S., O’Connor D.J., Mittnacht S., Zhong S., Lu X. (1999). RB regulates the stability and the apoptotic function of p53 via MDM2. Mol. Cell.

[66] Saha S., Bhattacharjee P., Guha D., Kajal K., Khan P., Chakraborty S., Mukherjee S., Paul S., Manchanda R., Khurana A. (2015). Sulphur alters NFκB-p300 cross-talk in favor of p53–p300 to induce apoptosis in non-small cell lung carcinoma. Int. J. Oncol..

[67] Kawashima H., Takatori H., Suzuki K., Iwata A., Yokota M., Suto A., Minamino T., Hirose K., Nakajima H. (2013). Tumor suppressor p53 inhibits systemic autoimmune diseases by inducing regulatory T cells. J. Immunol..

[68] Park J.S., Lim M.A., Cho M.L., Ryu J.G., Moon Y.M., Jhun J.Y., Byun J.K., Kim E.K., Hwang S.Y., Ju J.H. (2013). p53 controls autoimmune arthritis via STAT-mediated regulation of the Th17 cell/Treg cell balance in mice. Arthritis Rheum..

[69] Thomasova D., Mulay S.R., Bruns H., Anders H.J. (2012). p53-independent roles of MDM2 in NF-κB signaling: Implications for cancer therapy, wound healing, and autoimmune diseases. Neoplasia.

[70] Simelyte E., Rosengren S., Boyle D.L., Corr M., Green D.R., Firestein G.S. (2005). Regulation of arthritis by p53: Critical role of adaptive immunity. Arthritis Rheum..

[71] Leech M., Xue J.R., Dacumos A., Hall P., Santos L., Yang Y., Li M., Kitching A.R., Morand E.F. (2008). The tumour suppressor gene p53 modulates the severity of antigen-induced arthritis and the systemic immune response. Clin. Exp. Immunol..

[72] Okuda Y., Okuda M., Bernard C.C. (2003). Regulatory role of p53 in experimental autoimmune encephalomyelitis. J. Neuroimmunol..

[73] Zheng S.J., Lamhamedi-Cherradi S.E., Wang P., Xu L., Chen Y.H. (2005). Tumor suppressor p53 inhibits autoimmune inflammation and macrophage function. Diabetes.

[74] Zhang S., Zheng M., Kibe R., Huang Y., Marrero L., Warren S., Zieske A.W., Iwakuma T., Kolls J.K., Cui Y. (2011). Trp53 negatively regulates autoimmunity via the STAT3-Th17 axis. FASEB J..

[75] Fierabracci A. (2011). Recent insights into the role and molecular mechanisms of the autoimmune regulator (AIRE) gene in autoimmunity. Autoimmun. Rev..

[76] Sakaguchi S., Yamaguchi T., Nomura T., Ono M. (2008). Regulatory T cells and immune tolerance. Cell.

[77] Cools N., van Tendeloo V.F., Smits E.L., Lenjou M., Nijs G., van Bockstaele D.R., Berneman Z.N., Ponsaerts P. (2008). Immunosuppression induced by immature dendritic cells is mediated by TGF-β/IL-10 double-positive CD4+ regulatory T cells. J. Cell Mol. Med..

[78] Collison L.W., Workman C.J., Kuo T.T., Boyd K., Wang Y., Vignali K.M., Cross R., Sehy D., Blumberg R.S., Vignali D.A. (2007). The inhibitory cytokine IL-35 contributes to regulatory T cell function. Nature.

[79] Pandiyan P., Zheng L., Ishihara S., Reed J., Lenardo M.J. (2007). CD4+CD25+Foxp3+ regulatory T cells induce cytokine deprivation-mediated apoptosis of effector CD4+ T cells. Nat. Immunol..

[80] Onishi Y., Fehervari Z., Yamaguchi T., Sakaguchi S. (2008). Foxp3+ natural regulatory T cells preferentially form aggregates on dendritic cells in vitro and actively inhibit their maturation. Proc. Natl. Acad. Sci. USA.

[81] Krammer P.H., Arnold R., Lavrik I.N. (2007). Life and death in peripheral T cells. Nat. Rev. Immunol..

[82] Green D.R., Droin N., Pinkoski M. (2003). Activation-induced cell death in T cells. Immunol. Rev..

[83] Lenardo M.J. (1991). Interleukin-2 programs mouse alpha beta T lymphocytes for apoptosis. Nature.

[84] Boehme S.A., Lenardo M.J. (1996). TCR-mediated death of mature T lymphocytes occurs in the absence of p53. J. Immunol..

[85] Singh N., Yamamoto M., Takami M., Seki Y., Takezaki M., Mellor A.L., Iwashima M. (2010). CD4(+)CD25(+) regulatory T cells resist a novel form of CD28- and Fas-dependent p53-induced T cell apoptosis. J. Immunol..

[86] Watanabe M., Moon K.D., Vacchio M.S., Hathcock K.S., Hodes R.J. (2014). Downmodulation of tumor suppressor p53 by T cell receptor signaling is critical for antigen-specific CD4+ T cell responses. Immunity.

[87] Minton K. (2014). p53 controls the crowd. Nat. Rev. Immunol..

[88] Lowe J.M., Menendez D., Bushel P.R., Shatz M., Kirk E.L., Troester M.A., Garantziotis S., Fessler M.B., Resnick M.A. (2014). p53 and NF-κB coregulate proinflammatory gene responses in human macrophages. Cancer Res..

[89] Muñoz-Fontela C., Macip S., Martinez-Sobrido L., Brown L., Ashour J., Garcia-Sastre A., Lee S.W., Aaronson S.A. (2008). Transcriptional role of p53 in interferon-medaited antiviral immunity. J. Exp. Med..

[90] Taura M., Eguma A., Suico M.A., Shuto T., Koga T., Komatsu K., Komune T., Sato T., Saya H., Li J.-D. (2008). p53 regulates Toll-like receptor 3 expression and function in huamn epithelial cell lines. Mol. Cell. Biol..

[91] Youlyouz-Marfak I., Gachard N., le Clorennec C., Najjar I., Baran-Marszak F., Reminieras L., May E., Bornkamm G.W., Fagard R., Feuillard J. (2008). Identification of a novel p53-dependent activation pathway of STAT1 by antitumour genotoxic agents. Cell Death Differ..

[92] Dirisina R., Katzman R.B., Goretsky T., Managlia E., Mittal N., Williams D.B., Qiu W., Yu J., Chandel N.S., Zhang L. (2011). p53 and PUMA independently regulate apoptosis of intestinal epithelial cells in patients and mice with colitis. Gastroenterology.

[93] Herkel J., Kam N., Erez N., Mimran A., Heifetz A., Eisenstein M., Rotter V., Cohen I.R. (2004). Monoclonal antibody to a DNA-binding domain of p53 mimics charge structure of DNA: Anti-idiotypes to the anti-p53 antibody are anti-DNA. Eur. J. Immunol..

[94] Hara T., Ogawa F., Muroi E., Komura K., Takenaka M., Hasegawa M., Fujimoto M., Sato S. (2008). Anti-p53 autoantibody in systemic sclerosis: Association with limited cutaneous systemic sclerosis. J. Rheumatol..

[95] Kovacs B., Patel A., Hershey J.N., Dennis G.J., Kirschfink M., Tsokos G.C. (1997). Antibodies against p53 in sera from patients with systemic lupus erythematosus and other rheumatic diseases. Arthritis Rheum..

[96] Shiau M.Y., Kuo T.M., Tsay G.J., Chiou H.L., Lee Y.L., Chang Y.H. (2002). Absence of anti-p53 antibodies in Chinese patients with rheumatoid arthritis and systemic lupus erythematosus: Comment on the concise communication by Kovacs et al.. Arthritis Rheum..

[97] Chauhan R., Handai R., Das T.P., Pati U. (2004). Over-expression of TATA binding protein (TBP) and p53 and autoantibodies to these antigens are features of systemic sclerosis, systemic lupus erythematosus and overlap syndromes. Clin. Exp. Immunol..

[98] Mimura Y., Yazawa N., Tamaki Z., Ashida R., Jinnin M., Asano Y., Tada Y., Kubo M., Ihn H., Tamaki K. (2007). Anti-p53 antibodies in patients with dermatomyositis/polymyositis. Clin. Rheumatol..

[99] Herkel J., Mimran A., Erez N., Kam N., Lohse A.W., Märker-Hermann E., Rotter V., Cohen I.R. (2001). Autoimmunity to the p53 protein is a feature of systemic lupus erythematosus (SLE) related to anti-DNA antibodies. J. Autoimmun..

[100] Kuhn H.M., Kromminga A., Flammann H.T., Frey M., Layer P., Arndt R. (1999). p53 autoantibodies in patients with autoimmune diseases: A quantitative approach. Autoimmunity.

[101] Achiron A., Feldman A., Magalashvili D., Dolev M., Gurevich M. (2012). Suppressed RNA-polymerase 1 pathway is associated with benign multiple sclerosis. PLoS ONE.

[102] Firestein G.S., Echeverri F., Yeo M., Zvaifler N.J., Green D.R. (1997). Somatic mutations in the p53 tumor suppressor gene in rheumatoid arthritis synovium. Proc. Natl. Acad. Sci. USA.

[103] Yamanishi Y., Boyle D.L., Rosengren S., Green D.R., Zvaifler N.J., Firestein G.S. (2002). Regional analysis of p53 mutations in rheumatoid arthritis synovium. Proc. Natl. Acad. Sci. USA.

[104] Han Z., Boyle D.L., Shi Y., Green D.R., Firestein G.S. (1999). Dominant-negative p53 mutations in rheumatoid arthritis. Arthritis Rheum..

[105] Volodko N., Salla M., Eksteen B., Fedorak R.N., Huynh H.Q., Baksh S. (2015). TP53 codon 72 Arg/Arg polymorphism is associated with a higher risk for inflammatory bowel disease development. World J. Gastroenterol..

[106] Egiziano G., Bernatsky S., Shah A.A. (2016). Cancer and autoimmunity: Harnessing longitudinal cohorts to probe the link. Best Pract. Res. Clin. Rheumatol..

[107] Giani C., Fierabracci P., Bonacci R., Gigliotti A., Campani D., de Negri F., Cecchetti D., Martino E., Pinchera A. (1996). Relationship between breast cancer and thyroid disease: Relevance of autoimmune thyroid disorders in breast malignancy. J. Clin. Endocrinol. Metab..

[108] Shah A.A., Casciola-Rosen L. (2015). Cancer and scleroderma: A paraneoplastic disease with implications for malignancy screening. Curr. Opin. Rheumatol..

[109] Jung D.J., Jin D.H., Hong S.W., Kim J.E., Shin J.S., Kim D., Cho B.J., Hwang Y.I., Kang J.S., Lee W.J. (2010). Foxp3 expression in p53-dependent DNA damage responses. J. Biol. Chem..

[110] Katchman B.A., Barderas R., Alam R., Chowell D., Field M.S., Esserman L.J., Wallstrom G., LaBaer J., Cramer D.W., Hollingsworth M.A. (2016). Proteomic mapping of p53 immunogenicity in pancreatic, ovarian, and breast cancers. Proteom. Clin. Appl..

[111] Garziera M., Montico M., Bidoli E., Scalone S., Sorio R., Giorda G., Lucia E., Toffoli G. (2015). Prognostic value of serum antibody immunity to p53 oncogenic protein in ovarian cancer: A systematic review and a meta-analysis. PLoS ONE.

[112] Chai Y., Peng B., Dai L., Qian W., Zhang Y., Zhang J.Y. (2014). Autoantibodies response to MDM2 and p53 in the immunodiagnosis of esophageal squamous cell carcinoma. Scand. J. Immunol..

